# Deficiency in ST6GAL1, one of the two α2,6-sialyltransferases, has only a minor effect on the pathogenesis of prion disease

**DOI:** 10.3389/fmolb.2022.1058602

**Published:** 2022-11-14

**Authors:** Natallia Makarava, Elizaveta Katorcha, Jennifer Chen-Yu Chang, Joseph T. Y. Lau, Ilia V. Baskakov

**Affiliations:** ^1^ Center for Biomedical Engineering and Technology, University of Maryland School of Medicine, Baltimore, MD, United States; ^2^ Department of Anatomy and Neurobiology, University of Maryland School of Medicine, Baltimore, MD, United States; ^3^ Department of Molecular and Cellular Biology, Roswell Park Comprehensive Cancer Center, Buffalo, NY, United States

**Keywords:** prion, prion diseases, N-glycosylation, sialic acid, sialyltransferases, ST6GAL1, ST6GAL2, mouse

## Abstract

Prion diseases are a group of fatal neurodegenerative diseases caused by misfolding of the normal cellular form of the prion protein or PrP^C^, into a disease-associated self-replicating state or PrP^Sc^. PrP^C^ and PrP^Sc^ are posttranslationally modified with N-linked glycans, in which the terminal positions occupied by sialic acids residues are attached to galactose predominantly *via* α2-6 linkages. The sialylation status of PrP^Sc^ is an important determinant of prion disease pathogenesis, as it dictates the rate of prion replication and controls the fate of prions in an organism. The current study tests whether a knockout of ST6Gal1, one of the two mammalian sialyltransferases that catalyze the sialylation of glycans *via* α2-6 linkages, reduces the sialylation status of PrP^Sc^ and alters prion disease pathogenesis. We found that a global knockout of ST6Gal1 in mice significantly reduces the α2-6 sialylation of the brain parenchyma, as determined by staining with *Sambucus Nigra* agglutinin. However, the sialylation of PrP^Sc^ remained stable and the incubation time to disease increased only modestly in *ST6Gal1* knockout mice (ST6Gal1-KO). A lack of significant changes in the PrP^Sc^ sialylation status and prion pathogenesis is attributed to the redundancy in sialylation and, in particular, the plausible involvement of a second member of the sialyltransferase family that sialylate *via* α2-6 linkages, ST6Gal2.

## Introduction

Prion diseases are a group of fatal neurodegenerative diseases in humans and animals that can arise spontaneously or *via* transmission (Prusiner, 2001). Prions or PrP^Sc^ are transmissible and spread from host-to-host or cell-to-cell by recruiting and converting the cellular form of the prion protein or PrP^C^ into a disease-associated PrP^Sc^ state (Prusiner, 1997; Legname et al., 2004). Prions elicit multiple disease phenotypes characterized by different clinical symptoms, cell tropism, affected brain regions, deposition patterns and incubation times to disease (Collinge and Clarke, 2007). The diversity of disease phenotypes within the same host has been attributed to the ability of PrP^C^ to acquire multiple, structurally distinct, self-replicating PrP^Sc^ states referred to as prion strains (Bessen and Marsh, 1992; Safar et al., 1998; Peretz et al., 2001; Ayers et al., 2011; Gonzalez-Montalban et al., 2011; Klimova et al., 2015; Morales et al., 2016). The fact that prion strains are structurally different has been well documented (Caughey et al., 2022; Manka et al., 2022), yet how prion strains elicit multiple, strain-specific disease phenotypes has not been answered (Baskakov, 2021).

PrP^C^ is posttranslationally modified with one or two N-linked glycans and a GPI anchor (Stahl et al., 1987; Endo et al., 1989). At the terminal positions of the N-linked glycans are sialic acid residues that are attached to galactose predominantly *via* α2-6 or, less often, *via* α2-3 linkages (Turk et al., 1988; Endo et al., 1989; Stimson et al., 1999; Katorcha and Baskakov, 2017). Each of the two glycans can carry up to five sialic acid residues (Endo et al., 1989; Rudd et al., 1999). According to mass-spectroscopy analysis, the variation in structure and composition of N-linked glycans give rise to more than 400 different PrP^C^ sialoglycoforms (Endo et al., 1989; Stimson et al., 1999). Traditional mass-spectroscopy largely underestimates the sialylation status of glycans due to the stripping of sialic residues by acidic solvents, so the actual diversity is expected to be greater. As judged from biochemical analysis, up to 90% of the terminal positions of PrP^Sc^ N-glycans are sialylated (Katorcha and Baskakov, 2018). Upon conversion of PrP^C^ into PrP^Sc^, the sialylated glycans and GPI are carried over, giving rise to sialylated PrP^Sc^ (Bolton et al., 1985; Stahl et al., 1993; Rudd et al., 1999).

Among hundreds of PrP^C^ sialoglycoforms expressed in a brain, prion strains recruit sialoglycoforms selectively, according to the strain-specific structure of PrP^Sc^ and the fitness of individual PrP^C^ sialoglycoforms (Katorcha et al., 2015b; Baskakov and Katorcha, 2016). Previously, we proposed that as a result of selective recruitment, strain-specific patterns of carbohydrate groups are formed on PrP^Sc^ surfaces (Baskakov et al., 2018). Among solvent-exposed groups, terminal sialic acids and galactose residues are functionally most important, as they elicit response of innate immune cells (Srivastava et al., 2018). For instance, terminal galactose residues are sensed by myeloid cells and elicit an ‘eat-me’ response. Consistent with the above hypothesis, the sialylation status of the N-glycans of PrP^Sc^ was found to dictate the outcomes of prion infection (Katorcha et al., 2014; Srivastava et al., 2017). Partially desialylated PrP^Sc^, i.e. PrP^Sc^ with increased amounts of exposed galactose, failed to induce prion disease upon intracranial or intraperitoneal administration (Katorcha et al., 2014; Srivastava et al., 2017). Moreover, prion infectivity could be switched off and on in a reversible manner *via* removing and reinstalling the sialylation of PrP^Sc^, respectively (Katorcha et al., 2016a). Finally, desialylation of PrP^Sc^ altered its trafficking in the periphery, redirecting PrP^Sc^ away from secondary lymphoid organs to the liver (Srivastava et al., 2017).

Considering the import role of sialylation in dictating disease outcomes, modulating the sialylation status of PrP^Sc^ might offer new strategies for developing therapeutic intervention against prion diseases. Moreover, learning how carbohydrates dictate strain-specific pathology will help to establish the relationship between strain-specific structure and disease phenotype in a predictable manner. In mammals, sialylation of glycans takes place in the trans-Golgi and is executed by twenty sialyltransferases (STs) (Audry et al., 2011). STs are classified into four families according to the type of linkages synthesized (α2-3, α2-6, α2-8 or α2-9) and the selectivity towards N- or O-linked glycans (Takashima, 2008; Audry et al., 2011). Among the twenty STs, three enzymes (ST3Gal3, ST3Gal4 and ST3Gal6) sialylate N-linked glycans *via* an α2-3 linkage, whereas only two, ST6Gal1 and ST6Gal2, sialylate *via* an α2-6 linkage (Takashima, 2008; Audry et al., 2011). In PrP^Sc^, an α2-6 linkage is predominant (Endo et al., 1989; Katorcha and Baskakov, 2017), so we decided to target the ST6 family for manipulating the sialylation status of PrP^C^ and PrP^Sc^. Between the two members of the ST6 family, ST6Gal1 is believed to be the main enzyme responsible for α2-6 sialylation in mammals. In adults, it is expressed ubiquitously in almost all tissues, including the brain (Manzi et al., 2000; Martin et al., 2002). Numerous reports document the involvement of ST6GAL1-mediated sialylation in pathologies relating to inflammation, immune responses, hematopoietic regulation, and cancer (e.g. recently reviewed in (Gc et al., 2022). Very little is known of ST6GAL2 and its catalytic properties. In fact, nothing is known about the physiologic roles of ST6GAL2, and its expression appears to be restricted to a brain, lung and spleen (Krzewinski-Recchi et al., 2003; Laporte et al., 2009; Lehoux et al., 2010; Ohmi et al., 2021).

The current study tested whether a deficiency of ST6Gal1 reduces the sialylation status of PrP^Sc^ and alters prion disease pathogenesis. We found that a global knockout of ST6Gal1 significantly reduces α2-6 sialylation of the brain parenchyma, as judged from staining with *Sambucus Nigra* agglutinin (SNA). However, the sialylation of PrP^Sc^ remained stable and the incubation time to disease showed only a modest increase in ST6Gal1 knockout mice (ST6Gal1-KO). Lack of significant changes is attributed to the redundancy in sialylation of PrP^C^ and, in particular, the involvement of ST6GAL2. Our data indirectly points to the involvement of ST6GAL2, the only other known sialyltransferase to be able to construct the α2,6-Sia linkage in PrP, in the pathogenic progression of prion disease.

## Materials and methods

### Ethics statement

This study was carried out in strict accordance with the recommendations in the Guide for the Care and Use of Laboratory Animals of the National Institutes of Health. The animal protocol was approved by the Institutional Animal Care and Use Committee of the University of Maryland, Baltimore (Assurance Number A32000-01; Permit Number: 0215002).

### Animals

The ST6Gal1-KO mouse has a globally inactivated *ST6Gal1* gene and was originally produced by Marth and co-workers (Hennet et al., 1998). The strain was backcrossed >10 generations into the C57Bl/6 background. Age-matched C57Bl/6 animals were used as controls.

ST6Gal1-KO and C57BL/6 mice (females and males) were inoculated intracerebrally into the left hemisphere ∼2 mm to the left of the midline and ∼2 mm anterior to a line drawn between the ears with 20 μL of 1% 22L brain homogenate under isoflurane anesthesia. Inoculum is delivered slowly by a 26 G needle inserted to a depth of approximately 3 mm. Signs of neurological disease were detected between 127–138 days post inoculation and consisted of hind-limb clasp, ataxia, and weight loss. Within 13–26 days after the first clinical signs, mice became unable to walk on a beam, developed kyphosis and became lethargic. Mice were considered terminally ill when they were unable to rear and/or lost 20% of their weight. At this point they were euthanized by CO2 asphyxiation and decapitation.

### Western blot

10% (wt/vol) homogenates in PBS from brains and spleens were prepared using glass/Teflon homogenizers as described previously (Makarava et al., 2012). For analysis of brain-derived PrP^Sc^, an aliquot of 10% homogenate was diluted with nine volumes of 1% Triton X-100 in PBS, sonicated for 30 s inside a Misonix S-4000 microplate horn (Qsonica), and treated with 10 μg/ml of proteinase K (New England BioLabs) for 1 h at 37°C. For analysis of spleen-derived PrP^Sc^, 10% homogenate was supplemented with 1% Triton X-100, sonicated as above, and treated with 20 μg/ml of proteinase K for 1 h at 37°C. Resulting brain and spleen samples were supplemented with 4xSDS loading buffer and heated for 10 min in a boiling water bath. Samples were loaded onto NuPAGE 12% Bis-Tris gels, transferred to PVDF membranes, and probed with anti-prion antibodies ab3531 (Abcam, Waltham, MA). Western blot signals were visualized using the FlourChem M imaging system (Protein Simple, San Jose, CA). For generating glycoform profiles and calculating total PrP^Sc^ amount, densitometry analysis of 1D blots was performed using the “Lane profile” and “Multiplex Band Analysis” functions in AlphaView software (Protein Simple), respectively.

### 2D electrophoresis

For 2D electrophoresis of brain-derived materials, an aliquot of 10% (wt/vol) homogenate was diluted with nine volumes of 1% (vol/vol) Triton X-100 in PBS, sonicated as above, and treated with 25 μg/ml of proteinase K for 30 min at 37 °C. For 2D electrophoresis of spleen-derived material, 250 µL of 10% (wt/vol) homogenate was diluted 1:1 with PBS, aliquotted into 0.2-ml thin-wall PCR tubes, sonicated as above, and combined into one tube, which was subjected to a 30-min 16,000 g centrifugation at 4°C. The pellet was resuspended in 25 µL of 1% (wt/vol) Triton X-100 in PBS, treated with 20 μg/ml of proteinase K for 30 min at 37 °C, then supplemented with 4xSDS loading buffer, heated for 10 min in a boiling water bath, and processed for 2D electrophoresis. 25 µL of the resulting samples were solubilized for 1 h at room temperature in 200 µL of solubilization buffer (8 M Urea, 2% (wt/vol) CHAPS, 5 mM TBP, 20 mM TrisHCl pH 8.0), then alkylated by adding 7 µL of 0.5 M iodoacetamide and incubated for 1 h at room temperature in the dark. Then, 1150 µL of ice-cold methanol was added and samples were incubated for 2 h at −20°C. After 16,000 g centrifugation at 4°C, the supernatant was discarded, and the pellet was re-solubilized in 160 µL rehydration buffer (7 M urea, 2 M thiourea, 1% (wt/vol) DTT, 1% (wt/vol) CHAPS, 1% (wt/vol) Triton X-100, 1% (vol/vol) ampholyte, and a trace amount of Bromophenol Blue). Fixed immobilized pre-cast IPG strips (cat. # ZM0018, Life Technologies, Carlsbad, CA) with a linear pH gradient 3–10 were rehydrated in 155 µL of the resulting mixture overnight at room temperature inside IPG Runner cassettes (cat. # ZM0008, Life Technologies). Isoelectrofocusing (first dimension separation) was performed at room temperature with increasing voltage (175 V for 15 min, then 175–2,000 V linear gradient for 45 min, then 2,000 V for 30 min) on a Life Technologies Zoom Dual Power Supply using an XCell SureLock Mini-Cell Electrophoresis System (cat. # EI0001, Life Technologies). The IPG strips were then equilibrated for 15 min consecutively in 1) 6 M Urea, 20% (vol/vol) glycerol, 2% SDS, 375 mM Tris-HCl, pH 8.8, 130 mM DTT, then 2) 6 M Urea, 20% (vol/vol) glycerol, 2% SDS, 375 mM Tris-HCl, pH 8.8, 135 mM iodoacetamide, and loaded on 4–12% Bis-Tris ZOOM SDS-PAGE pre-cast gels (cat. # NP0330BOX, Life Technologies). For the second dimension, SDS-PAGE was performed for 1 h at 170 V. Immunoblotting was performed with ab3531 (Abcam).

### Histopathological study

Formalin-fixed brain halves divided at the midline (left hemisphere) were treated in formic acid (95%) to deactivate prion infectivity before being embedded in paraffin. 4 µm sections mounted on slides were processed for hematoxylin-eosin (H&E) staining and immunohistochemistry. To expose epitopes, slides were subjected to 20 min hydrated autoclaving at 121°C in trisodium citrate buffer, pH 6.0 with 0.05% Tween 20. For detection of disease-associated PrP, a 5 min treatment with 88% formic acid was used following autoclaving. PrP was stained with anti-prion antibody SAF-84 (Cayman Chemical, Ann Arbor, MI). Rabbit anti-Iba1 (Wako, Richmond, VA) was used to stain microglia. Detection was performed using 3,3’ Diaminobenzidine (DAB) Quanto chromogen and substrate (VWR, Radnor, PA).

For SNA staining, 4 µm brain sections mounted on slides were subjected to the standard rehydration procedure, then submerged in 10 mM tri-sodium citrate buffer, pH 6.0, boiled for 5 min by microwaving at 20% power, and cooled for 1 h before proceeding with lectin staining. Incubation in 3% hydrogen peroxide in methanol for 20 min was used to remove endogenous peroxidase activity. After a 5 min wash in running water, slides were incubated for 1 h with 5 μg/ml biotin-labeled elderberry bark lectin (SNA, Vector laboratories, Burlingame, CA) diluted in lectin buffer, pH 7.6 (50 mM Tris, 150 mM NaCl, 1 mM MgCl2, 0.75 mM CaCl2). Following three 5 min washes in lectin buffer, the slides were incubated for 30 min in 5 μg/ml horse radish peroxidase-labeled streptavidin (Thermo Fisher scientific, Waltham, MA), then again washed three times with lectin buffer, and developed with DAB Quanto chromogen and substrate (VWR, Radnor, PA).

### RT-qPCR

Brains were divided at the midline, and the right hemispheres were used to dissect cortex, hippocampus, midbrain and thalamus regions. 10% (wt/vol) homogenates were prepared within 1.5 ml tubes in Trizol (Thermo Fisher Scientific, Waltham, MA, United States), using RNase-free disposable pestles (Fisher scientific, Hampton, NH). Total RNA was isolated using the Aurum Total RNA Mini Kit (Bio-Rad, Hercules, CA, United States) and subjected to DNase I digestion to remove contaminating genomic DNA. Total RNA was dissolved in elution buffer and stored at −80°C. An absorbance 260/280 value of ∼2.0, determined using a NanoDrop ND-1000 Spectrophotometer (Thermo Fisher Scientific, Waltham, MA, United States) proved RNA purity. Reverse transcription was performed using 1 μg of extracted RNA and an iScript cDNA Synthesis Kit (Bio-Rad, Hercules, CA, United States). RT-qPCR was performed in triplicate from one ST6Gal1-KO and one C57Bl/6 (WT) brain using the SsoAdvanced universal SYBR Green Supermix (Bio-Rad, Hercules, CA, United States) with Bio-Rad designed and validated primers for ST6Gal1 (qMmuCID0009827). Alternative ST6Gal1 primers designed for exon 2 were as follows: forward 5′- ACA​CCA​CTG​AAT​GGG​AGG​GT - 3′, and reverse 5′- CAG​AGA​TCC​TGC​AGA​AGA​CAC​G - 3’. The housekeeping gene TBP (Bio-Rad, qMmuCID0040542) was used for normalization. The PCR protocol consisted of a treatment at 95 °C for 2 min followed by 40 amplification cycles with the following steps: 95 °C for 5 s, and 60 °C for 30 s. The RT-qPCR was performed with a CFX96 Touch Real-Time PCR Detection System (Bio-Rad, Hercules, CA, United States), and analyzed with Bio-Rad CFX Manager software (Bio-Rad, Hercules, CA, United States).

## Results

### The knockout of *ST6Gal1* significantly reduces α2-6 sialylation of the brain parenchyma

**FIGURE 1:**
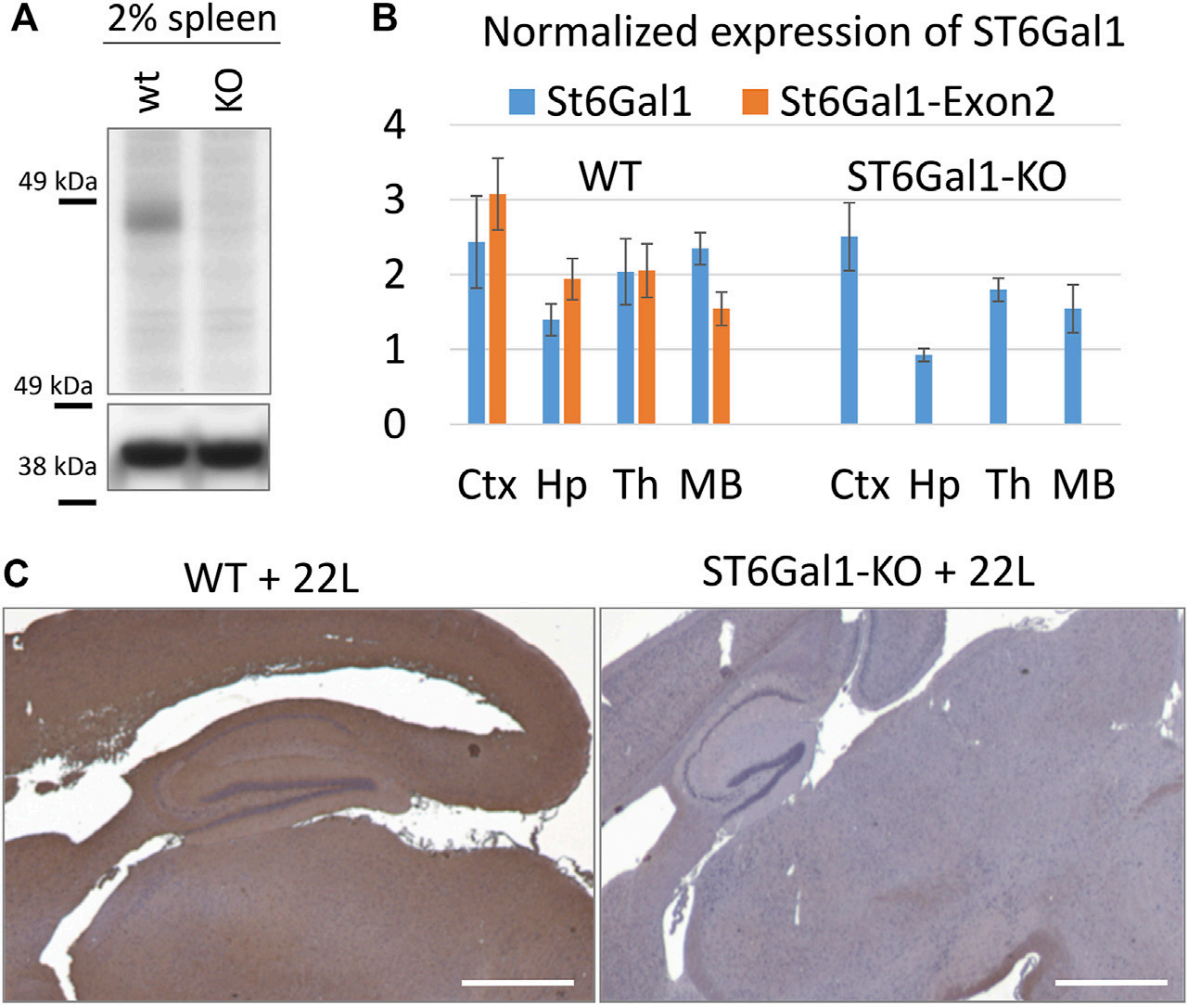
The global knockout of ST6Gal1. **(A)** Western blot of 2% spleen tissues from ST6Gal1-KO (KO) and control C57Bl/6 (WT) mice stained with anti-ST6Gal1 antibody (#AF5924, R&D Systems, Minneapolis, MN). **(B)** RT-qPCR analysis of ST6Gal1 expression using Bio-Rad primers (qMmuCID0009827) or primers to Exon 2 of the gene in cortex (Ctx), hippocampus (Hp), thalamus (Th) and midbrain (MB) from ST6Gal1-KO (KO) and control C57Bl/6 (WT) mice. Each sample was analyzed in triplicate. The mean and standard deviation are shown. TBP was used as a housekeeping gene. **(C)** Staining of 22L-infected brains of ST6Gal1-KO (KO) and C57Bl/6 (WT) mice, euthanized at 146 and 145 days post-inoculation (dpi), respectively, using *Sambucus Nigra* lectin (SNA). Scale bar = 1 mm.

In mice, the highest level of ST6Gal1 expression was found in the spleen (Takashima et al., 2002). In the current study, spleens were used to confirm the global knockout of *ST6Gal1*. As expected, ST6Gal1was found in control C56Bl/6 mice (WT) but was lacking in ST6Gal1-KO mice (Figure 1A). The level of ST6Gal1 expression in the brains of WT mice was below the detection limits of the Western blot (data not shown). To confirm the knockout of *ST6Gal1* in animal brains, we used RT-qPCR with two sets of *ST6Gal1* primers, including primers to the exon 2. ST6Gal1-KO mice were generated by deleting the exon 2 that resulted in a loss of over 50% of the *ST6Gal1* coding sequence including cytoplasmic, transmembrane, and considerable catalytic domain sequences (Hennet et al., 1998). As expected, RT-qPCR with primers to exon 2 detected expression of *ST6Gal1* in multiple brain areas of WT mice, but not in ST6Gal-KO mice (Figure 1B).

To test whether the knockout of *ST6Gal1* reduces sialylation of the brain parenchyma, brain slices were stained with SNA lectin that detects terminal α2-6 sialylation of both N- and O-linked glycans. Consistent with previous studies (Martin et al., 2002), ST6Gal1-KO mice showed significant loss in α2-6 sialylation of the brain parenchyma across multiple brain regions including thalamus, hippocampus and cortex in comparison to control WT mice (Figure 1C).

### Knockout of *ST6Gal1* had a modest impact on the incubation time to disease

**FIGURE 2:**
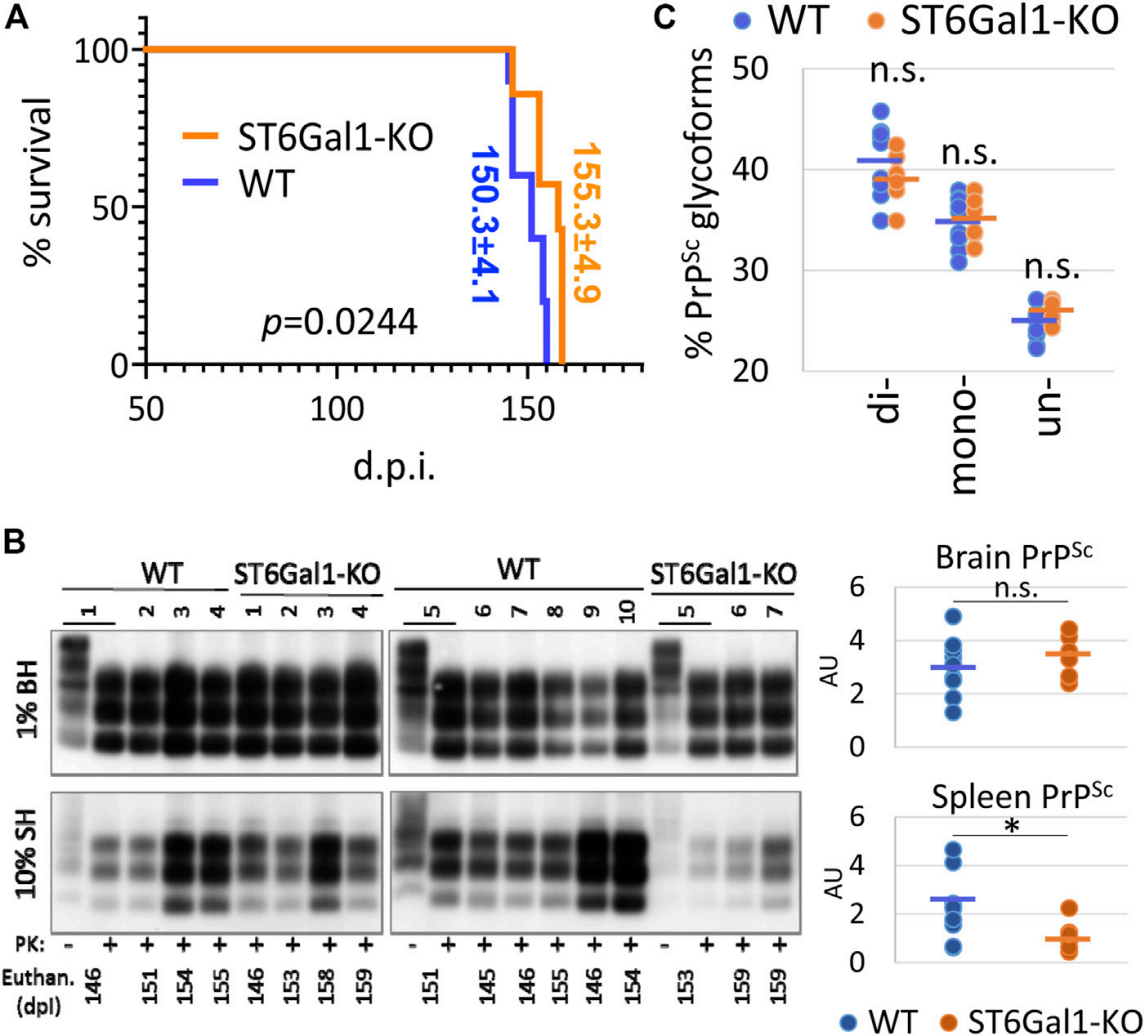
A modest impact of ST6Gal1 knockout on the incubation time to disease. **(A)** Kaplan-Meier survival plot for ST6Gal1-KO and C57Bl/6 (WT) mice inoculated IC with 1% scrapie brain homogenate of the 22L strain. *p* value was calculated using Mantel-Cox test for comparison of survival curves. **(B)** Western blot analysis and quantification of PrP^Sc^ in brain homogenates (BH) and spleen homogenates (SH) of ST6Gal1-KO and C57Bl/6 (WT) mice inoculated with 22L. Samples were treated with PK. Untreated (-PK) samples were used as a reference, and were loaded as a 10-fold dilution. Western blots were stained with the ab3531 antibody. PrP^Sc^ amount in brains and spleens were compared using unpaired *t*-tests and presented as individual animals and means (*n* = 10 WT, n = 7 ST6Gal1-KO, *p < 0.05). **(C)** The percentage of di-, mono- and unglycosylated brain-derived PrP^Sc^ from ST6Gal1-KO and C57Bl/6 (WT) mice inoculated with 22L. Data presented as individual animals and means (*n* = 10 WT and n = 7 ST6Gal1-KO).

**FIGURE 3:**
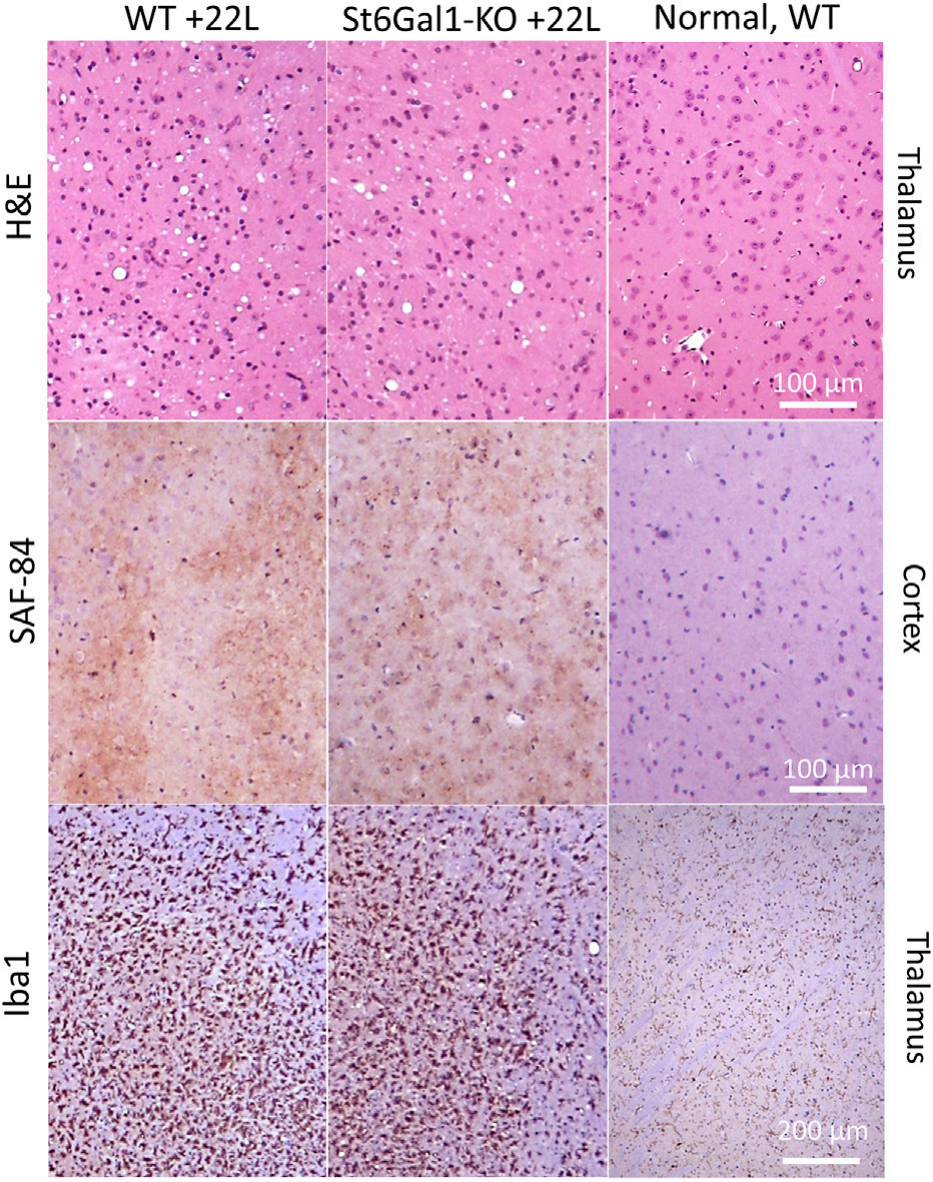
Histopathological analysis of 22L-infected mouse brains. Vacuolation revealed with hematoxylin-eosin (H&E) staining, deposition of PrP^Sc^ stained with SAF-84 antibody, and reactive microglia stained for Iba1 in ST6Gal1-KO and C57Bl/6 (WT) mice inoculated IC with 22L and euthanized at 146 and 145 dpi, respectively. Staining of normal C57Bl/6 (WT) euthanized at 212 days of age is shown as references (right panels).

To test whether the knockout of *ST6Gal1* modifies prion disease pathogenesis, ST6Gal1-KO and control WT mice were inoculated intracerebrally with 1% scrapie brain homogenate of 22L, a mouse-adapted prion strain. ST6Gal1-KO and control WT groups succumbed to the diseases at 155.3 ± 4.9 and 150.3 ± 4.1 days post-inoculation, respectively (Figure 2A). While ST6Gal1-KO mice showed slightly longer incubation times relative to the control group, the difference in their survival curves was bordering statistical significance (Figure 2A). No statistically significant difference was found between the two groups with respect to the amounts of PK-resistant PrP^Sc^ in their brains (Figure 2B). The knockout of *ST6Gal1* did not change the ratios of un-, mono-, and diglycosylated glycoforms within PrP^Sc^ (Figure 2C). As reported previously (Katorcha et al., 2015b), in the 22L mouse adapted strain, the diglycosylated glycoform is predominant, whereas the unglycosylated is the least abundant PrP^Sc^ glycoform. In both groups, PrP^Sc^ spread to spleen showing large variations between individual animals with respect to its amounts, which is expected for the 22L strain inoculated intracranially (Figure 2B). In spleen, the amounts of PrP^Sc^ was lower in ST6Gal1-KO group relative to WT group (Figure 2B). Histopathological analysis showed the main pathological hallmarks of prion disease including PrP^Sc^ deposition, spongiosis and reactive microglia in both ST6Gal1-KO and control WT mice (Figure 3).

### A *ST6Gal1* knockout did not affect the sialylation status of PrP^Sc^ in the brain or spleen

**FIGURE 4:**
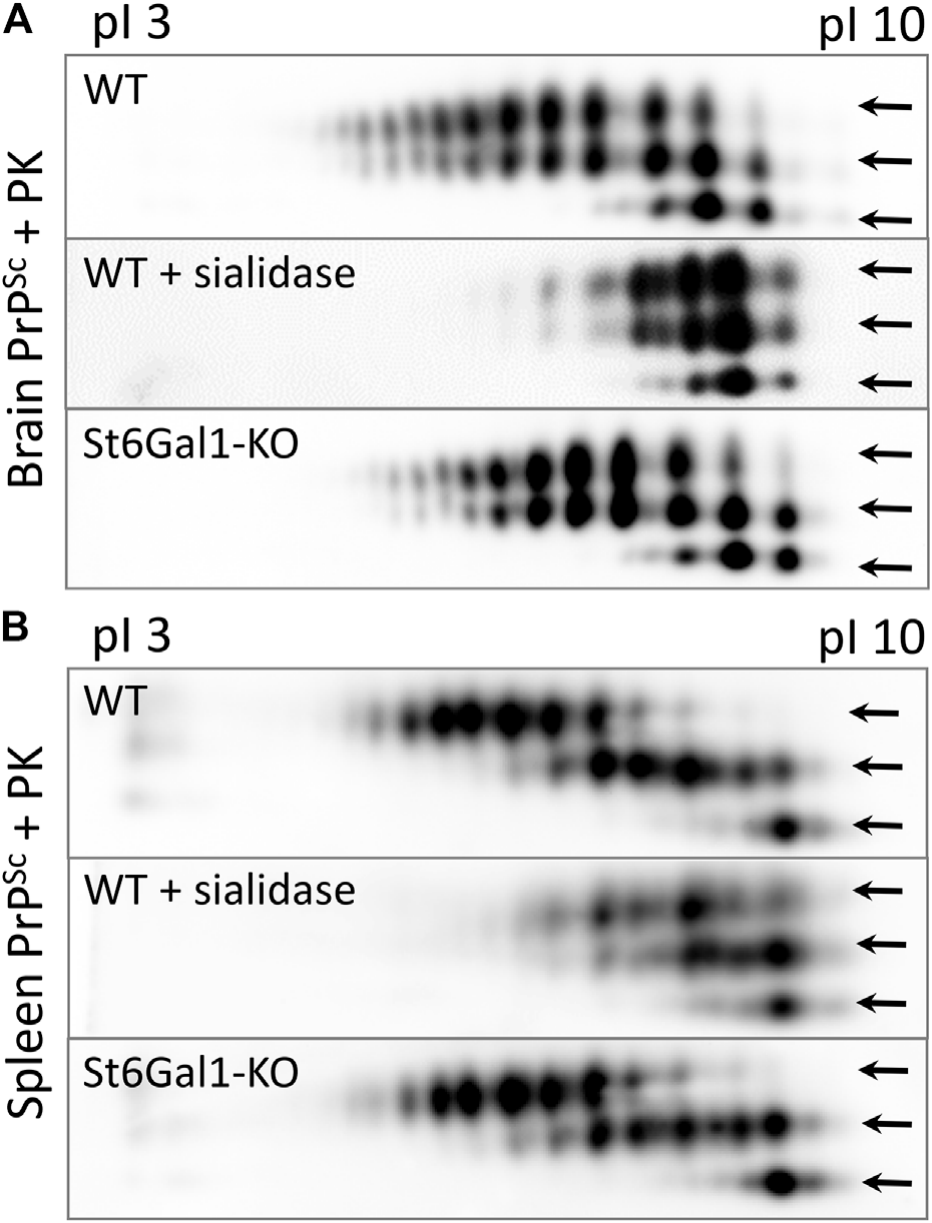
Analysis of the sialylation status of PrP^Sc^. Representative 2D Western blot of brain-derived PrP^Sc^ from ST6Gal1-KO and C57Bl/6 (WT) mice euthanized at 146 and 145 dpi, respectively **(A)**, and spleen-derived PrP^Sc^ from ST6Gal1-KO and C57Bl/6 (WT) mice euthanized at 158 and 154 dpi, respectively **(B)**. Mice were inoculated IC with 1% scrapie brain homogenate of the 22L strain. Middle panels show brain- and spleen-derived PrP^Sc^ from WT mice desialylated using sialidase *Arthrobacter ureafaciens*. Prior to 2D blot, brain and spleen materials were treated with 20 μg/mL PK. Anti-PrP antibody ab3531 was used for immunodetection. Arrows point at di-, mono- and non-glycosylated glycoforms.

To test whether the knockout of *ST6Gal1* reduces the sialylation status of PrP^Sc^, brain- and spleen-derived material was analyzed using two-dimensional (2D) gel electrophoresis. Prior to 2D analysis, the samples were treated with PK and then denatured, so that the sialylation status of individual PrP molecules could be visualized as a distribution of charge isoforms in the horizontal dimension of 2D. Each sialic acid residue adds one negative charge and hypersialylated PrP molecules move toward an acidic pH, whereas hyposialylated PrP molecules move toward a basic pH in the horizontal dimension of 2D gels (Figure 4). Three horizontal rows of charged isoforms from bottom to top correspond to the non-, mono- and diglycosylated PrP molecules.

Consistent with previous studies, non-glycosylated PrP molecules showed multiple charge isoforms (all of which were at basic pH) that were attributed to the structural heterogeneity of GPI anchors (Katorcha et al., 2016b). No notable differences between ST6Gal1-KO and control group brains with respect to the distribution of PrP sialoglycoforms can be found in brain- or spleen-derived PrP^Sc^ (Figures 4A,B). As reported in previous studies (Srivastava et al., 2015), the sialylation pattern of brain-derived PrP^Sc^ was different from that of spleen-derived PrP^Sc^ (Figures 4A,B). As expected, treatment with sialidase *Arthrobacter ureafaciens* (AU), that has broad substrate specificity, shifted the distribution of PrP sialoglycoforms to basic pH (Figures 4A,B). These results illustrate that the global knockout of *ST6Gal1* did not change the sialylation pattern of PrP^Sc^ in the brain or spleen.

## Discussion

Considering the important role of sialylation in dictating the rate of prion replication and their fate in an organism, manipulation of the sialylation status of PrP^Sc^ *via* altering the steady-state sialylation of its precursor PrP^C^ might offer a novel strategy for developing therapeutic intervention against prion diseases. Sialylation of glycoproteins is controlled by two groups of enzymes: sialyltransferases (STs) and neuraminidases or sialidases (Audry et al., 2011; Miyagi and Yamaguchi, 2012). Previously, we found that a knockout of *Neu1*, *Neu3*, *Neu4*, or both *Neu3* and *Neu4*, which are all expressed in the brain, does not affect the steady-state levels of PrP^C^ sialylation in mice (Katorcha et al., 2015a). This fact suggests that desialylated PrP^C^ molecules do not contribute to the steady-state level of PrP^C^ sialoglycoforms, presumably, due to degradation. In the absence of detectable changes in PrP^C^ sialylation in *Neu’s* knockout models, targeting STs seemed to offer a more effective strategy for manipulating the sialylation levels of PrP^C^ and PrP^Sc^. In support of this notion, the sialylation levels of PrP^C^ in cultured N2a cells were suppressed to a large degree by the administration of a general inhibitor of STs, 3F_ax_-Neu5Ac (Katorcha et al., 2015a). However, because 3F_ax_-Neu5Ac targets a broad spectrum of STs and blocks global sialylation (Burkart et al., 2000; Lairson et al., 2008), it causes irreversible kidney dysfunction in animals which leads to rapid death (Macauley et al., 2014). In the absence of inhibitors that selectively target individual STs (Wang et al., 2016; Szabo and Skropeta, 2017), we decided to test the effect of a knockout of *ST6Gal1*.

Western blotting of spleen tissues from the ST6Gal1-KO cohort confirmed a global knockout of *ST6Gal1*, while RT-qPCR documented the lack of *ST6Gal1* expression in brain tissue. Consistent with previous studies (Martin et al., 2002), SNA staining revealed significantly lower levels of α2-6 sialylation of the brain parenchyma in ST6Gal1-KO mice relative to that of control WT animals. However, despite a global knockout of the *ST6Gal1* gene, the sialylation status of brain- and spleen-derived PrP^Sc^ did not show notable changes. In spleen-derived PrP^Sc^, dyglycosylated PrP molecules shifted toward acidic pH relative to those of monoglycosylated PrP molecules in both WT and ST6Gal1 mice (Figure 4B), suggesting that spleen-derived PrP^Sc^ is fully sialylated. Such sialylation pattern is consistent with very high levels of sialyltransferase activity in secondary lymphoid organs. In contrast, in brain-derived PrP^Sc^ of WT and ST6Gal1, the pattern of charged isoforms of diglycosylated PrP molecules was very similar to that of monoglycosylated PrPs (Figure 4A). Such pattern suggests that in a brain, diglycosylated PrP molecules are only partially sialylated and on average have a similar number of sialic acid residues per PrP molecule as monoglycosylated PrPs, despite the fact that diglycosylated PrPs have two glycans, while monoglycosylated PrPs have only one glycan. Nevertheless, similar sialylation levels of PrP^Sc^ in ST6Gal1-KO and WT mice could be due to 1) the possibility that sialylation is conducted primarily by the ST6Gal2 enzyme, 2) redundancy in activities of ST6Gal1 and ST6Gal2 enzymes, and/or 3) redundancy between the ST6 and ST3 families of STs.

As the last member of STs discovered, *ST6Gal2* has not been characterized as thoroughly as ST6Gal1 (Krzewinski-Recchi et al., 2003). ST6Gal2 seems to exhibit a more narrow enzymatic substrate specificity in comparison to ST6Gal1 (Takashima et al., 2002; Takashima et al., 2003). While the *ST6Gal1* gene is expressed in almost all tissues, the expression of *ST6Gal2* shows a restricted tissue-specific pattern and is primarily limited to the brain and spleen (Krzewinski-Recchi et al., 2003; Laporte et al., 2009; Lehoux et al., 2010; Ohmi et al., 2021). A direct comparison of expression using RT-qPCR revealed that in an adult mouse brain, *ST6Gal1* and *ST6Gal2* genes are expressed at similar levels (Ohmi et al., 2021). However, *ST6Gal1* mRNAs were found across different cell types in mice including neurons, astrocytes and microglia, whereas the expression of *ST6Gal2* gene was detected primarily in astrocytes and neurons. Notably, the expression of *STI6Gal2* in primary epithelial cultures was found to be upregulated by treatment with IL-6, suggesting that *STI6Gal2* responds to pro-inflammatory conditions (Laporte et al., 2009; Lehoux et al., 2010). In a mouse brain, region-specific expression of the *ST6Gal2* gene and the region-specific sialylation status of PrP^Sc^ showed the same ranking order (Makarava et al., 2020a), raising the possibility that ST6Gal2 is responsible for the sialylation. It would be interesting to test whether a knockout of *ST6Gal2* or a double knockout of *ST6Gal1* and *ST6Gal2* affect prion disease pathogenesis.

It is not surprising that in the absence of notable effects on PrP^Sc^ sialylation, the knockout of *ST6Gal1* caused only a modest delay in the incubation time to terminal disease. Because in brain *ST6Gal1* is expressed across different cell types, the delay could be attributed to a perturbation of the homeostatic states of neurons and glia or changes in their response to prion infection. For example, ST6Gal1 was found to promote long-term cell activation through two pro-inflammatory pathways, NFκB and JAK/STAT3 (Holdbrooks et al., 2020). STAT3 is a transcription factor which is believed to be a master regulator of astrocyte reactivity (Ben Haim et al., 2015; Ceyzériat et al., 2018; Reichenbach et al., 2019). Because reactive astrocytes associated with prion disease are detrimental to neurons (Kushwaha et al., 2021; Makarava et al., 2021), the delay in the incubation time could be attributed to an attenuation of a STAT3-mediated activation of astrocytes in ST6Gal1-KO mice. Several families of receptors including siglecs, selectins, galectins, complement system components and asialoglycoprotein receptors recognize terminal sialic acid or galactose residue, and are involved in innate immunity (Linnartz et al., 2012; Rabinovich and Croci, 2012). As a result of a significant reduction in α2-6 sialylation of the brain parenchyma in ST6Gal1-KO mice, one should expect a perturbation of multiple signaling pathways involved in innate immunity (Zhuo and Bellis, 2011). Alternatively, deficiency in *ST6Gal1* could affect the incubation time by altering homeostatic and/or reactive states of peripheral immune cells involved in prion disease pathogenesis (Nasirikenari et al., 2006; Liu et al., 2011). ST6Gal1 was found to regulate monocyte-macrophage development and survival (Rusiniak et al., 2022). As such, a *ST6Gal1* deficiency could attenuate pro-inflammatory toxic effects of monocytes infiltrating prion-infected brains. Lower amounts of PrP^Sc^ in spleens of ST6Gal1-KO mice relative to WT mice is consistent with the idea that ST6Gal1 regulates function of peripheral immune cells important for prion trafficking in periphery and/or prion replication in secondary lymphoid organs.

Understanding the role of sialylation might be important for gaining insight into the etiology of sporadic CJD. There is a decline in the total sialic acid content as well as cell surface sialylation with normal aging (Svennerholm et al., 1997). Previously, we showed that the rate of PrP^Sc^ replication is accelerated by PrP^C^ with low sialylation status (Katorcha et al., 2014; Katorcha et al., 2015b). It is not known whether a possible increase in the proportion of hyposialylated PrP^C^ with aging might facilitate the spontaneous conversion of PrP^C^ into PrP^Sc^ and increase the rate of sporadic CJD (Baskakov and Katorcha, 2016). Consistent with this hypothesis is a correlation between the region-specific sialylation status of PrP^Sc^ and the vulnerability of brain regions to prion infection (Makarava et al., 2020a). The thalamus displays prion deposition and chronic neuroinflammation prior to cortex and hippocampus and, by the terminal stage of the disease, the thalamus is affected more severely than any other brain region (Karapetyan et al., 2009; Sandberg et al., 2014; Carroll et al., 2016; Makarava et al., 2019; Makarava et al., 2020b; Makarava et al., 2021). Regardless of prion strain, thalamic PrP^Sc^ is sialylated less than cortex and hippocampus PrP^Sc^ (Makarava et al., 2020a). Lower levels of sialylation promotes faster prion replication and triggers profound neuroinflammation (Katorcha et al., 2015b; Srivastava et al., 2018). As such, testing a knockout of *ST6Gal2* in future studies might provide new insight into etiology of sporadic CJD.

## Data Availability

The original contributions presented in the study are included in the article/Supplementary Material, further inquiries can be directed to the corresponding author.
